# Effect of Functional Interleukin-10 Polymorphism on Pegylated Interferon-α Plus Ribavirin Therapy Response in Chronic Hepatitis C Virus Patients Infected With 3a Genotype in Pakistani Population

**DOI:** 10.5812/hepatmon.10274

**Published:** 2013-06-08

**Authors:** Muhammad Sohail Afzal, Sadia Anjum, Najam Us Sahar Sadaf Zaidi

**Affiliations:** 1 Atta ur Rahman School of Applied Biosciences, National University of Science and Technology, Islamabad, Pakistan

**Keywords:** Hepacivirus, Interleukin-10, Polymorphism, Genetic, Pakistan

*Dear Editor,*

Pakistan is a low socio economic country having more than 10 million people infected with hepatitis C Virus (HCV) with a major genotype of 3a (GT 3a) (1). Due to high rate of resistance to standard Interferon plus Ribavirin therapy, it is highly needed to identify new marker for response prediction to therapy. Interleukin 10 (IL-10) is a key member of Cytokine, which regulates Th1/Th2 Cytokine balance, a major part of immune system against infection (2). IL-10 production varies inter individually based on functional polymorphism (-1082 G/A, -819 C/T and -592 C/A) in its promoter region. Previously we have shown that IL-10 polymorphic variants play important role in HCV susceptibility/prevention (2). Recently we conducted a study to analyze the impact of functionally important IL-10 polymorphism on outcomes of standard Interferon-α plus Ribavirin therapy. The results of current study strengthen previous findings that IL-10 polymorphism effect disease susceptibility in Pakistan (2-4). Our results showed that high IL-10 producing -1082 GG genotype (P = 0.02; OR = 0.4; 95% CI = 0.2-0.8) and GTA haplotype (P = 0.03; OR = 0.55; 95% CI = 0.3-1) were significantly higher in HCV patients as compared to healthy subjects, while IL-10 -1082 GA genotype (P = 0.03; OR = 1.95; 95% CI = 1.1-3.4) showed protective effect against HCV infection. The current data failed to show any significant corelation between IL-10 polymorphism inheritance and standard therapy response in HCV patients. Age and pretreatment viral load are the parameters which influence therapy response; Patients with sustained virological response (SVR) were younger and had lower pretreatment HCV RNA levels. No gender-based statistical difference was found between chronic hepatitis C patients who achieved SVR or failed to respond. To our knowledge this is the first report from Pakistan to evaluate the role of IL-10 polymorphism on the outcomes of standard antiviral therapy. We are unable to find any corelation between IL-10 polymorphic variants and interferon therapy outcomes, may be due to small number of subjects. This study is a preliminary and is on small scale. We believe that our effort may stimulate some additional personal genetic makeup based studies on larger scale using these and new multi-locus analysis approaches for a deeper analysis of the epistatic interaction of the pro- and anti -inflammatory molecules toward hepatitis C progression. Better understanding of genetic factors that have effect on hepatitis C progression and pathogeneses will provide scientific basis for the development of new immunomodulatory treatments for chronic hepatitis C patients and will also help health care workers about the standard therapy effectiveness.
